# Preliminary Evaluation of Rapid Visual Identification of *Burkholderia pseudomallei* Using a Newly Developed Lateral Flow Strip-Based Recombinase Polymerase Amplification (LF-RPA) System

**DOI:** 10.3389/fcimb.2021.804737

**Published:** 2022-01-18

**Authors:** Jin Li, Qiu Zhong, Mei-Yun Shang, Min Li, Yuan-Su Jiang, Jia-Jun Zou, Shan-Shan Ma, Qing Huang, Wei-Ping Lu

**Affiliations:** ^1^ Department of Laboratory Medicine, Daping Hospital, Army Medical University (Third Military Medical University), Chongqing, China; ^2^ Department of Laboratory Medicine, Ministry of Education (M.O.E.) Key Laboratory of Laboratory Medicine Diagnostics, Chongqing Medical University, Chongqing, China

**Keywords:** *Burkholderia pseudomallei*, LF-RPA assay, 16S rRNA gene sequencing analysis, real-time PCR, *orf2* gene

## Abstract

*Burkholderia pseudomallei* is an important infectious disease pathogen that can cause melioidosis. Melioidosis is mainly prevalent in Thailand, northern Australia and southern China and has become a global public health problem. Early identification of *B. pseudomallei* is of great significance for the diagnosis and prognosis of melioidosis. In this study, a simple and visual device combined with lateral flow strip-based recombinase polymerase amplification (LF-RPA) was developed, and the utility of the LF-RPA assay for identifying *B. pseudomallei* was evaluated. In order to screen out the optimal primer probe, a total of 16 pairs of specific primers targeting the *orf2* gene of *B. pseudomallei* type III secretion system (T3SS) cluster genes were designed for screening, and F1/R3 was selected as an optimal set of primers for the identification of *B. pseudomallei*, and parameters for LF-RPA were optimized. The LF-RPA can be amplified at 30-45°C and complete the entire reaction in 5-30 min. This reaction does not cross-amplify the DNA of other non-*B. pseudomallei* species. The limit of detection (LOD) of this assay for *B. pseudomallei* genomic DNA was as low as 30 femtograms (fg), which was comparable to the results of real-time PCR. Moreover, 21 clinical *B. pseudomallei* isolates identified by 16S rRNA gene sequencing were retrospectively confirmed by the newly developed LF-RPA system. Our results showed that the newly developed LF-RPA system has a simple and short time of operation and has good application prospect in the identification of *B. pseudomallei.*

## Introduction

*Burkholderia pseudomallei* is a gram-negative and facultative intracellular bacterium belonging to *Burkholderia*. It can cause melioidosis, a severe infectious disease primarily affecting humans and other animals (Wiersinga et al., 2018; Chakravorty and Heath, 2019; Gassiep et al., 2020). *B. pseudomallei* can enter the body through skin mucosal infiltration or aerosol inhalation (Saxena et al., 2019). Melioidosis is mainly prevalent in Thailand, northern Australia and southern China and has become a global public health problem (Limmathurotsakul et al., 2016; Mohan et al., 2017). Currently, there is no licensed melioidosis vaccine available for humans or animals. If not diagnosed and treated promptly, melioidosis can lead to sepsis and high mortality (Wang et al., 2020).

In the genus *Burkholderia*, the morphology and biochemical characteristics of *B. pseudomallei* is very similar to that of other *Burkholderiaceae* isolates, and it is difficult to distinguish them (Duval et al., 2014; Peddayelachagiri et al., 2016; Titball et al., 2017). Therefore, identifying *B. pseudomallei* and other closely related *Burkholderiaceae* isolates is a considerable challenge for clinical microbiology laboratories (Thibault et al., 2004). The culture-based method is the gold standard for identifying *B. pseudomallei*, but it usually takes 3-5 days; therefore, it is easy to miss the optimal time for treatment (Lee et al., 2005; Choi et al., 2020). The ELISA method can be used to detect the specific antibodies to *B. pseudomallei* (Kohler et al., 2016; Suttisunhakul et al., 2016; Yatsomboon et al., 2021). However, due to the high rate of positive antibodies in these epidemic areas, the diagnostic value of this method is poor (Hii et al., 2017; Yatsomboon et al., 2021). Molecular biology methods such as conventional PCR and real-time PCR have been widely used in the identification of *B. pseudomallei* (Supaprom et al., 2007; Kaestli et al., 2012). However, they currently have many disadvantages; for example, these methods have cumbersome operation steps, are easily contaminated, are very time-consuming, require expensive equipment and require skilled personnel (Pal et al., 2018; Li et al., 2019a; Wong Tzeling et al., 2021). Therefore, research on a new method that can quickly and accurately identify *B. pseudomallei* is needed.

Recombinase polymerase amplification (RPA) is an emerging isothermal amplification technique that does not require skilled personnel or lengthy and complicated procedures. Labeled amplification can be easily detected by using lateral flow strips (Jiang et al., 2020a; Lalremruata et al., 2020; Wu et al., 2020). The RPA system consists of three main proteins: recombinase, single-stranded DNA binding protein (SSB), and DNA polymerase (Juma et al., 2021). Amplification is initiated by a primer-recombinase complex that invades the DNA double strand on the homologous sequence of the primer. SSB then stabilizes the reaction and the polymerase begins to stretch (Xue et al., 2020). The RPA reaction is completed in 8-10 min at 37°C, making it an ideal technique for point-of-care testing (Ma et al., 2019; Yu et al., 2019). In addition, the amplification of markers can be detected by lateral flow (LF) strips, and the results can be easily read with the naked eye (Jiang et al., 2020b). However, aerosol contamination caused by unenclosed operation is a bottleneck problem that limits the application of lateral flow strip-based recombinase polymerase amplification (LF-RPA). Therefore, an airtight, miniaturized and portable detection device combined with LF-RPA urgently needs to be developed. In the present study, we attempted to introduce a device combined with LF-RPA and evaluated the utility of the LF-RPA assay for identifying *B. pseudomallei*.

## Materials and Methods

### Bacterial Strains and Genomic DNA Preparation

A total of 50 clinical isolates, including twenty-one *B. pseudomallei*, four *B. thailandensis*, three *B. multivorans*, two *B. cenocepacia*, four *B. cepacia*, four *P. aeruginosa*, four *E. coli*, four *K. pneumoniae* and four *A. baumannii* isolates, were collected from Hainan Hospital, Daping Hospital and Southwest Hospital (**Supplementary Table 1**). Genomic DNA of all these isolates was extracted using a TIANGEN genomic DNA isolation kit (TIANGEN, Beijing, China) in accordance with the manufacturer’s instructions. The DNA samples were stored at -80°C until use. Due to the limited opportunity to obtain the international standard strain of *B. pseudomallei*, we selected *B. pseudomallei* BPC006 as the positive control strain (Fang et al., 2012).

### Identification of Isolates by Gene Sequencing Analysis

All isolates in this study were confirmed by 16S rRNA gene sequencing (**Supplementary Figure 1**). The conventional PCR products were sent to Beijing Genomics Institute (BGI) Biotechnology Corporation for sequencing, and the sequencing results were searched against the GenBank database using the BLAST algorithm (http://www.ncbi.nlm.nih.gov/blast) (Li et al., 2019a).

### Primer and Probe Design

The primers for basic RPA and probe for LF-RPA were designed to target the *orf2* gene of *B. pseudomallei* type III secretion system (T3SS) cluster genes (Rainbow et al., 2002). The primers and probes were designed manually in the conused region of *orf2* gene according to the design principles of RPA primers and probes. Primer-BLAST of NCBI was used to confirm the specificity of the primers, and online OligoEvaluator software (http://www.oligoevaluator.com) was used to analyze the potential of primer dimers and hairpins (Xue et al., 2020). All primers and probes were synthesized and purified by BGI Biotechnology Corporation using high-performance liquid chromatography (HPLC).

### Basic RPA Assay

The basic RPA reaction was achieved by the TwistAmp Basic kit (TwistDx, U.K.). The reaction contained 29.5 µL reaction buffer, 11.2 µL double-distilled water, 2.4 µL forward primer (10 μM), 2.4 µL reverse primer (10 μM), 2 µL DNA template, and 2.5 µL 280 mM magnesium acetate. The mixture was vortexed and spun short and then incubated at 40°C for 15 min. The RPA product was purified by phenol (Solarbio, Beijing, China) and analyzed on a 1% agarose gel. A total of 16 pairs of specific primers were tested by of basic RPA, and the best efficiency primers were chosen for LF-RPA. A FAM-labeled probe was designed for LF-RPA according to the description of the TwistAmp nfo kit (TwistDx, U.K.) (Li et al., 2019b). In this study, we selected *B. pseudomallei* BPC006 as the positive control, double distilled water as the negative control.

### LF-RPA Assay and a Newly Developed LF-RPA System

The LF-RPA reaction included 29.5 µL of reaction buffer, 11.2 µL of double-distilled water, 2.1 µL of forward primer (10 µM), 2.1 µL of reverse primer (10 µM), 0.6 µL of the probe (10 µM), 2 µL of DNA template and 2.5 µL of 280 mM magnesium acetate (El Wahed et al., 2021). In this study, we developed a simple and visual device combined with LF-RPA.

As **Supplementary Figure 2** shows, 2 µL of DNA template was added to the premixed RPA reaction buffer. Then, the reaction tube was incubated in a simple heating device at 40°C for 10 min. Thereafter, for analysis by lateral flow, the labeled amplification was diluted at 1:50 in PBS. A HybriDetect 1 lateral flow strip (Milenia Biotech, Germany) was placed into test tubes containing diluted amplicon until the test line of the positive control was visible. The amplification results were interpreted according to visual observation of the test line and control line. With this newly developed system, the whole process could be completed in less than 15 min, from the time the DNA sample entered the device to when the reaction result was interpreted, without aerosol contamination.

### Optimization of Temperature and Time for LF-RPA Assay

To find the optimal amplification temperature, LF-RPA assay was performed using 1.5 ng genomic DNA (gDNA) at various temperature settings from 25 to 50°C for 20 min. Experiments with different reaction times (5-35 min) were then monitored at 40°C. The experiment was carried out in a single reaction and repeated independently three times.

### Sensitivity and Specificity of the LF-RPA Assay

To evaluate the sensitivity of LF-RPA, we diluted the gDNA of *B. pseudomallei* with double distilled water to obtain the final concentration of 3×10^2^ pg- 3 fg per reaction. The labeled amplifiers were diluted with PBS and HybriDetect 1 lateral flow strip was used for visual detection, as described above. In order to get the actual LOD, 30 fg gDNA of *B. pseudomallei* were tested 20 times by LF-RPA. To verify the specificity of LF-RPA, gDNA of *B. thailandensis*, *B. cepacia*, *P. aeruginosa*, *E. coli*, *K. pneumoniae*, and *A. baumannii* (0.5-2.0 ng) was examined to identify possible cross-reactions. The experiment was repeated three times with the same result.

### Real-Time PCR Assay

To compare their sensitivities, the diluted DNA samples of *B. pseudomallei* were tested in parallel by an established real-time PCR protocol at 95°C for 30 s, followed by 40 cycles of 95°C for 5 s and 60°C for 30 s. The reaction included 2.0 µL of DNA template, 12.5 µL of Premix Ex Taq (Probe qPCR) (2X), 8.5 µL of double-distilled water, 0.5 µL of forward primer (10 µM), 0.5 µL of reverse primer (10 µM), and 1.0 µL of the probe (10 µM). Identification was performed on a CFX96 real-time PCR detection system (Bio-Rad). A threshold cycle (Ct value) <38 was determined as the positive sample as described by Supaprom (Supaprom et al., 2007). In order to get the actual LOD, 30 fg gDNA of *B. pseudomallei* were tested 20 times by real-time PCR.

### Identification of Clinical Isolates by a Newly Developed LF-RPA System

To further explore the diagnostic potential of the newly developed LF-RPA system for on-site application, the gDNA of 50 clinical isolates was chosen to verify the feasibility of this system. The performance of the newly developed system was compared to that of 16S rRNA gene sequencing.

## Results

### Bacterial Identity

All 50 clinical isolates were retrospectively confirmed by a complete 16S rRNA gene sequencing analysis, including twenty-one *B. pseudomallei*, four *B. thailandensis*, three *B. multivorans*, two *B. cenocepacia*, four *B. cepacia*, four *P. aeruginosa*, four *E. coli*, four *K. pneumoniae* and four *A. baumannii*, as shown in **Supplementary Figure 1**.

### Design and Screening of Primers

**Figure 1:**
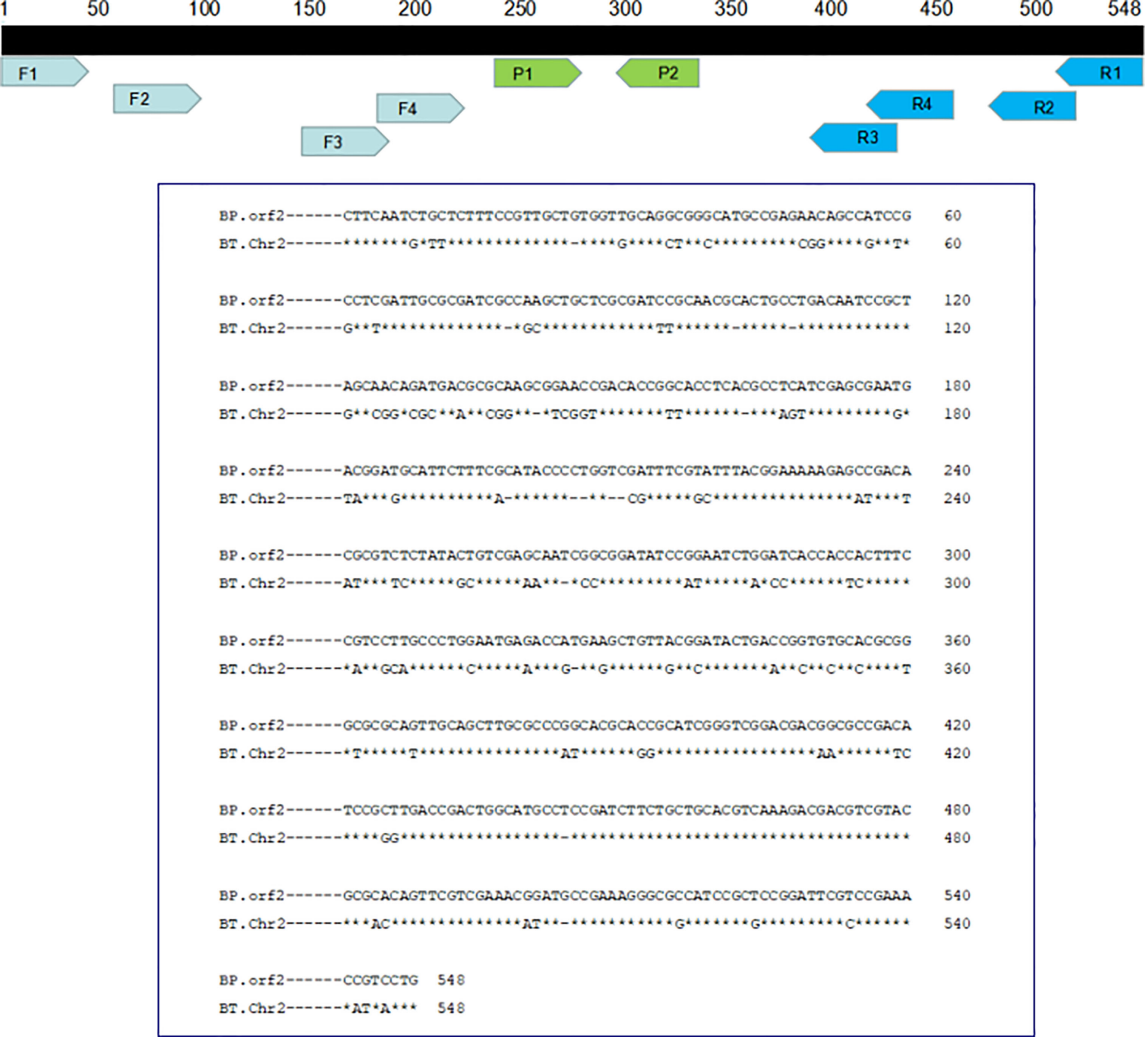
Relative positions of the amplicon targets on the *B. pseudomallei orf2* gene. The nucleotide sequence of *B*. *pseudomallei*-specific target DNA is shown together with the locations of primers and probes and their names. *B*. *pseudomallei*: GenBank Accession No. AF074878; *B*. *thailandensis*: GenBank Accession No. CP008785.1.

**Figure 2:**
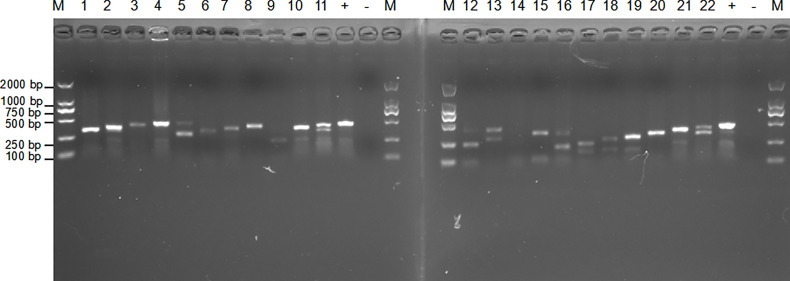
Primer screening for basic RPA. A total of 16 pairs of specific primers targeting the *orf2* gene of *B*. *pseudomallei* type III secretion system (T3SS) cluster genes were designed for screening by basic RPA (2 ng gDNA as template, at 40°C for 15 min). Lanes 1-16 are primers F1/R4, F1/R3, F1/R2, F1/R1, F2/R4, F2/R3, F2/R2, F2/R1, F3/R4, F3/R3, F3/R2, F3/R1, F4/R4, F4/R3, F4/R2, and F4/R1. Lanes 17-22 are the results of repeated electrophoresis of some sets of primers. Lane + is the positive control. Lane - is the negative control. M: DNA marker.

The primers were manually designed within the conserved regions of the *orf2* gene of *B. pseudomallei* type III secretion system (T3SS) cluster genes according to the principles of RPA primer design (**Figure 1** and **Table 1**). As shown in **Figure 2**, the F1/R4, F1/R3, F1/R1 and F3/R3 amplicons exhibited the best efficiency for the basic RPA reaction. Considering the length of the amplicon, F1/R3 was selected and subjected to further experiments in our study. The forward primer for LF-RPA was unlabeled, while the reverse primer for LF-RPA was conjugated with biotin at the 5’ end. The probe used for the LF-RPA assay was a 46 bp length of nucleotides with FAM labeled at the 5’ end, a tetrahydrofuran residue site (THF, also referred to as a dSpacer) 30 nucleotides downstream of the 5’ end and a block group (C3spacer) at the 3’ end (Saxena et al., 2019). The nucleotide sequences of the forward primer, the reverse primer and the probe used in the study are shown in **Table 1**. The relative positions of primers and probes on the *orf2* gene are depicted in **Figure 1**.

**Table 1:** Primers and probe used in the present study.

Assay	Name	Sequence (5´-3´) and modification	Length (bp)
Basic RPA	orf2-F1	CTTCAATCTGCTCTTTCCGTTGCTGTGG	28
	orf2-F2	CTCGATTGCGCGATCGCCAAGCTGCTCGCG	30
	orf2-F3	CTCACGCCTCATCGAGCGAATGACGGATG	29
	orf2-F4	CATACCCCTGGTCGATTTCGTATTTACGG	29
	orf2-R1	CAGGACGGTTTCGGACGAATCCGGAGCGG	29
	orf2-R2	TCGGCATCCGTTTCGACGAACTGTGCGCG	29
	orf2-R3	TCGTCTTTGACGTGCAGCAGAAGATCGGAG	30
	orf2-R4	TCGGAGGCATGCCAGTCGGTCAAGCGGATGTCG	33
LF-RPA	LF-F1	CTTCAATCTGCTCTTTCCGTTGCTGTGG	28
	LF-R3	[biotin] TCGTCTTTGACGTGCAGCAGAAGATCGGAG	29
	LF-P	[FAM]TCGAGCAATCGGCGGATATCCGGAATCTGG[THF]TCACCACCACTTTCCG[C3Spacer]	46
Real-time PCR	TTS1-F	CTTCAATCTGCTCTTTCCGTT	21
	TTS1-R	CAGGACGGTTTCGGACGAA	19
	TTS1-P	FAM-CCGGAATCTGGATCACCACCACTTTCC-BHQ	27

### Optimization of the Reaction Temperature and Time

**Figure 3:**
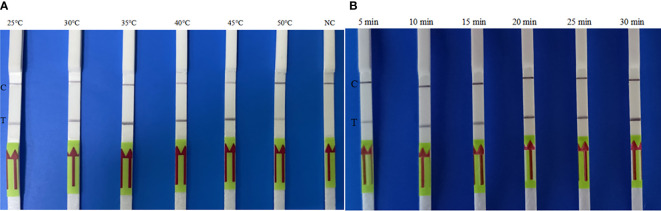
Optimization of the temperature **(A)** and time **(B)** for the LF-RPA. **(A)** gDNA of *B*. *pseudomallei* BPC006 (2 ng) was used in each reaction at the indicated temperatures for 10 min; **(B)** gDNA of *B*. *pseudomallei* BPC006 (2 ng) was used in each reaction at 40°C for the indicated time. NC, negative control; C, control line; T, test line. These experiments were repeated three times.

To assess the optimum amplification temperature, the LF-RPA assay was performed at the indicated temperatures for 20 min as recommended by the manufacturer. The best effect was achieved between 30°C and 45°C, and the test line on the strip could be observed over a wide temperature range (**Figure 3A**). The identification method is suitable for reaction in simple heating devices, so it can be used in any scale laboratory, community clinics and field environment. As shown in **Figure 3B**, the test band can be observed in an amplification time of as few as 5 min. As time goes on, the test lines become clearer. Considering the identification efficiency and sensitivity, an amplification time of 10 min is suitable for the LF-RPA assay. Therefore, the whole test, including RPA amplicon and strip-reading, took less than 15 min.

### Analytical Specificity of the LF-RPA Assay

**Figure 4:**
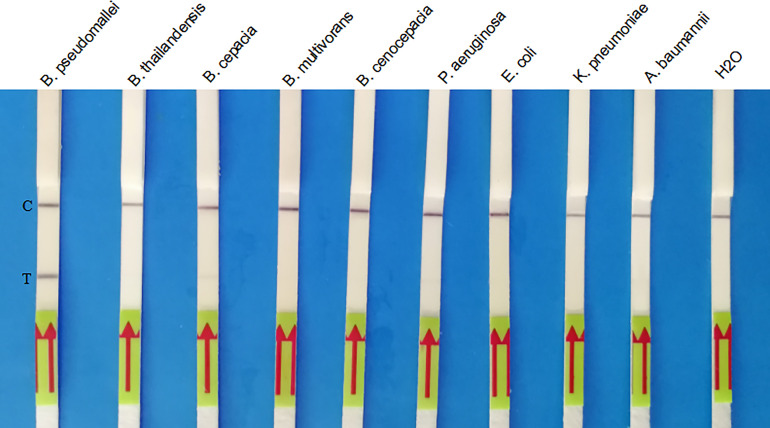
Specificity of the LF-RPA assay for the *orf2* gene of *B*. *pseudomallei* identification. Partial results showed that only the *B*. *pseudomallei* samples produced amplification signals, whereas the other pathogen samples and the negative control produced no amplification signals. This experiment was repeated three times with the same result.

As shown in **Figure 4**, only *B. pseudomallei* strain could produce positive results, while non-*B. pseudomallei* bacterial species could not. No cross-reactivity was observed for these clinical isolates (**Figure 4**), and the LF-RPA assay show high specificity (100%) for the identification of *B. pseudomallei*. A similar experiment was carried out three times with the same result.

### Analytical Sensitivity of the LF-RPA Assay

**Figure 5:**
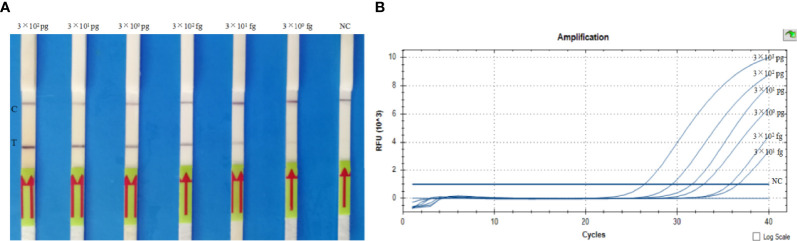
Sensitivity of the LF-RPA assay **(A)** and real-time PCR assay **(B)**. **(A)** Analytical sensitivity of the LF-RPA assay based on the quantity of genomic DNA of *B*. *pseudomallei* BPC006, serially diluted gDNA of *B*. *pseudomallei* (300 pg, 30 pg, 3 pg, 300 fg, 30 fg, 3 fg per reaction) was tested by LF-RPA at 40°C for 10 min. **(B)** Analytical sensitivity of the real-time PCR assay based on the quantity of genomic DNA of *B*. *pseudomallei* BPC006, serially diluted gDNA of *B*. *pseudomallei* (3 ng, 300 pg, 30 pg, 3 pg, 300 fg, 30 fg per reaction) was tested by real-time PCR at 95°C for 5 min, followed by 40 cycles of 95°C for 10 seconds and 60°C for 30 seconds. NC, negative control; C, control line; T, test line. This experiment was repeated three times with the same result.

The analytical sensitivity of the LF-RPA assay was assessed by adding 10-fold continuously diluted genomic DNA (300 pg, 30 pg, 3 pg, 300 fg, 30 fg, 3 fg per reaction) to a separate RPA reaction. The amplification products produced by the separation reaction were diluted as described above, and a HybriDetect 1 lateral flow strip was used to detect the amplification. The presence of test bands and control bands indicated a positive reaction, and only the presence of control bands indicated a negative reaction. LF-RPA can detect *B. pseudomallei* genomic DNA as low as 30 fg in each reaction (**Figure 5A**). LF-RPA has the same sensitivity as real-time PCR (**Figure 5B**). All the *orf2* gene of *B. pseudomallei* were tested positive by 20 tests.

### Feasibility of the Newly Developed LF-RPA System

Here, we further verified these strains by the newly developed LF-RPA system. Twenty-one of the strains were retrospectively identified as positive with respect to *B. pseudomallei*, which agreed with the 100% result of 16S rRNA gene sequencing (**Supplementary Table 1**). No cross-reaction was observed with other non-*B. pseudomallei* bacterial species, implying this system has a high specificity. The sensitivity and specificity were 100% for identifying *B. pseudomallei*. No significant differences between the identification results of the newly developed LF-RPA system and 16S rRNA gene sequencing were observed. The kappa value of the newly developed system was 1.0 (*P* < 0.001).

## Discussion

With the rapid development of nucleic acid detection technology, some diagnostic techniques based on PCR, such as conventional PCR and real-time PCR technology was used for rapid identification of *B. pseudomallei*. However, these methods rely on expensive equipment and require sophisticated operators. For some resource-poor laboratories, community clinic and field environment, these diagnostic techniques are not practical. In addition, the identification of *B. pseudomallei* using conventional PCR and real-time PCR will have problems such as low sensitivity and long detection process, which is not conducive to rapid detection and emergency detection. As an emerging isothermal amplification method for microbial identification, LF-RPA is superior to other methods because it has the advantages of saving time and being portable (Peng et al., 2019; Wu et al., 2019). Moreover, LF-RPA requires less equipment and laboratory conditions than real-time PCR and is generally costs less than real-time PCR. Based on these studies, we believe that LF-RPA is a fast, efficient and accurate method for the identification of *B. pseudomallei*.

The genome sequences of *B. pseudomallei* K96243 and *B. thailandensis* E264 obtained from NCBI were very similar, but the *orf2* gene of *B. pseudomallei* K96243 (GenBank accession no. AF074878) was different between the strains, indicating that this gene has high specificity in the identification of *B. pseudomallei*. Specific primers are important because they can influence the identification results of molecular diagnostic methods (Zhu et al., 2020). Previous studies have suggested that the *orf2* gene may distinguish *B. pseudomallei* from other non-*B. pseudomallei* bacterial species by real-time PCR (Supaprom et al., 2007). In the present study, a total of 16 pairs of specific primers targeting the *orf2* gene of *B. pseudomallei* type III secretion system (T3SS) cluster genes were designed for screening. After a series of basic RPA primers were tested, some LF-RPA primers showed visible bands in the expected size range, among which F1/R3 was the most efficient at 40°C for 15 min and was therefore chosen for follow-up experiments.

To evaluate the optimal amplification temperature, we evaluated the effect of temperature variations in the 25-50°C for LF-RPA amplification. No significant difference was observed at incubation temperatures ranging from 30-45°C, suggesting that reaction performance was not significantly affected over this temperature range (Fan et al., 2020). Therefore, we hypothesize that RPA amplification can be initiated by relatively low heat. With the extension of amplification duration from 5 to 30 min, the detection line of positive samples was enhanced. However, the distinction between positive and negative samples was evident regardless of whether the amplification occurred for 5 min or 30 min, revealing that the visual identification for RPA amplification had a high robustness (Yang et al., 2020). To shorten the whole detection time and ensure the detection efficiency and sensitivity, 10 min was utilized for LF-RPA amplification.

The LF-RPA assay developed in this study had a high species specificity that could detect all the *B. pseudomallei* isolates. In addition, there was no cross-reactivity with other non-*B. pseudomallei* bacteria species under the experimental conditions used, suggesting that LF-RPA has good specificity. Further studies should focus on verifying potential cross-reactivity with DNA from other *Burkholderia* isolates using the LF-RPA method developed herein. The developed LF-RPA assay was also highly sensitive and could detect 30 fg genomic DNA of *B. pseudomallei* per reaction, which was as good as that of real-time PCR.

To avoid aerosol contamination, a simple and visual device combined with LF-RPA has been developed. In detail, it was employed to handle the reaction tubes for on-site RPA amplification. Furthermore, the diagnostic potential of the newly developed LF-RPA system for on-site application was explored; 21 clinical *B. pseudomallei* isolates were retrospectively confirmed by the newly developed system, and 29 non-*B. pseudomallei* bacterial species showed negative results for *orf2* gene detection. Therefore, the positive detection rate of the newly developed system was 100%. There were no false positive results, indicating that the newly developed device is very practical. However, the limitation of this study is that the validation strains were limited in this study. To ensure the accuracy and reliability of the identification results, we need to further expand the number and types of validation strains to obtain better identification results (Zhao et al., 2021).

Moreover, this result demonstrates that the newly developed LF-RPA system is instantaneous, simple and reliable. Compared with the work of Peng et al., we proposed an alternative visual and noncontaminated detection method. Compared with other methods, detection by the lateral flow strip takes only 5 min and is easier to see. In addition, due to the extremely simplified operation process and the high robustness of RPA amplification, no trained operator is required. This approach can replace instrument-based approaches and provide a convenient solution, especially in resource-limited areas (El Wahed et al., 2021). This method can not only be used in the field screening of *B. pseudomallei*, but can be applied in many other fields, which has great potential for the poorly equipped diagnostic laboratory.

## Data Availability Statement

The datasets presented in this study can be found in online repositories. The names of the repository/repositories and accession number(s) can be found in the article/**Supplementary Material**.

## Author Contributions

JL, QZ, M-YS, and ML performed the laboratory measurements. QH and W-PL made substantial contributions to the conception and design. JL, Y-SJ, J-JZ, and S-SM participated in the experimental design and data analysis. JL drafted the manuscript. All authors read and approved the final manuscript.

## Funding

This work was financially supported by grants from the Military Medical Frontier Innovation Ability Training Program (No. 2019CXJSC018), Chongqing Medical Scientific Research Project (Joint Project of Chongqing Health Commission and Science and Technology Bureau) (No. 2020MSXM021), Chongqing Medical Scientific Research Project (Joint Project of Chongqing Health Commission and Science and Technology Bureau) (No. 2022QNXM034), Biosafety Construction Project (No. A3702022001) and University Outstanding Talent Support Program.

## Conflict of Interest

The authors declare that the research was conducted in the absence of any commercial or financial relationships that could be construed as a potential conflict of interest.

## Publisher’s Note

All claims expressed in this article are solely those of the authors and do not necessarily represent those of their affiliated organizations, or those of the publisher, the editors and the reviewers. Any product that may be evaluated in this article, or claim that may be made by its manufacturer, is not guaranteed or endorsed by the publisher.
