# Supplementary material for: Preliminary Evaluation of Rapid Visual Identification of Burkholderia pseudomallei Using a Newly Developed Lateral Flow Strip-Based Recombinase Polymerase Amplification (LF-RPA) System
Source: Front Cell Infect Microbiol. 2022 Jan 18;11:804737. doi: 10.3389/fcimb.2021.804737 (PMC8804217; doi:10.3389/fcimb.2021.804737)
Supplement: Supplementary file 1 [file DataSheet_1.doc]

**Title:** Preliminary evaluation of rapid visual identfication of *Burkholderia pseudomallei* using a newly developed lateral flow strip-based recombinase polymerase amplification (LF-RPA) system

**Authors:** Jin Li^1,2^, Qiu Zhong^1^, Mei-Yun Shang^1^, Min Li^1^, Yuan-Su Jiang^1^, Jia-Jun Zou^1^, Shan-Shan Ma^1^, Qing Huang^1*^, Wei-Ping Lu^1*^

^*^**Co-corresponding authors:**

Wei-Ping Lu, M.D. Ph.D., Email: [luweiping19710416@163.com](mailto:luweiping19710416@163.com)

Qing Huang, M.D. Ph.D., Email: [Q.Huang@gmail.com](mailto:Q.Huang@gmail.com)

Tel: +86-23-68757607

Fax: +86-23-68716530

^1^Department of Laboratory Medicine, Daping Hospital, Army Medical University (Third Military Medical University), Chongqing, 400042, P.R. China

^2^Department of Laboratory Medicine, M.O.E. Key Laboratory of Laboratory Medicine Diagnostics, Chongqing Medical University, Chongqing, 400016, P.R. China

**SUPPLEMENTARY MATERIALS INDEX**

**Supplementary Table 1. Characteristics of the Fifty isolates used for validation of the newly developed detection system.**

**Supplementary Figure 1. The *B. pseudomallei* isolate was retrospectively confirmed by a complete 16S rRNA gene sequencing analysis.**

**Supplementary Figure 2.** **Scheme of the instant and visual methods for identifying *B. pseudomallei* based on recombinase polymerase amplification.**

**Supplementary Table 1** Characteristics of the Fifty isolates used for validation of the newly developed detection system

| No. | Bacterial strain | Location | Isolation site | Concentration (ng/µL) | LF-RPA assay |
| --- | --- | --- | --- | --- | --- |
| 1 | *Burkholderia pseudomallei* | Daping Hospital | Sputum | 0.5 | + |
| 2 | *Burkholderia pseudomallei* | Daping Hospital | Blood | 0.5 | + |
| 3 | *Burkholderia pseudomallei* | Southwest Hospital | Blood | 0.6 | + |
| 4 | *Burkholderia pseudomallei* | Hainan Hospital | Sputum | 0.7 | + |
| 5 | *Burkholderia pseudomallei* | Hainan Hospital | Sputum | 0.6 | + |
| 6 | *Burkholderia pseudomallei* | Hainan Hospital | Blood | 0.8 | + |
| 7 | *Burkholderia pseudomallei* | Hainan Hospital | Sputum | 0.9 | + |
| 8 | *Burkholderia pseudomallei* | Hainan Hospital | Sputum | 0.8 | + |
| 9 | *Burkholderia pseudomallei* | Hainan Hospital | Blood | 1.6 | + |
| 10 | *Burkholderia pseudomallei* | Hainan Hospital | Sputum | 1.2 | + |
| 11 | *Burkholderia pseudomallei* | Hainan Hospital | BALF | 0.6 | + |
| 12 | *Burkholderia pseudomallei* | Hainan Hospital | BALF | 0.8 | + |
| 13 | *Burkholderia pseudomallei* | Hainan Hospital | Sputum | 1.2 | + |
| 14 | *Burkholderia pseudomallei* | Hainan Hospital | Sputum | 1.8 | + |
| 15 | *Burkholderia pseudomallei* | Hainan Hospital | Sputum | 2.0 | + |
| 16 | *Burkholderia pseudomallei* | Hainan Hospital | Sputum | 0.6 | + |
| 17 | *Burkholderia pseudomallei* | Hainan Hospital | Sputum | 0.6 | + |
| 18 | *Burkholderia pseudomallei* | Hainan Hospital | Sputum | 0.9 | + |
| 19 | *Burkholderia pseudomallei* | Hainan Hospital | Sputum | 0.5 | + |
| 20 | *Burkholderia pseudomallei* | Hainan Hospital | Sputum | 0.6 | + |
| 21 | *Burkholderia pseudomallei* | Hainan Hospital | BALF | 0.7 | + |
| 22 | *Burkholderia thailandensis* | Daping Hospital | Sputum | 0.6 | - |
| 23 | *Burkholderia thailandensis* | Daping Hospital | Blood | 0.6 | - |
| 24 | *Burkholderia thailandensis* | Daping Hospital | Sputum | 0.8 | - |
| 25 | *Burkholderia thailandensis* | Daping Hospital | Urine | 1.0 | - |
| 26 | *Burkholderia multivorans* | Daping Hospital | Sputum | 0.6 | - |
| 27 | *Burkholderia multivorans* | Daping Hospital | Sputum | 0.8 | - |
| 28 | *Burkholderia multivorans* | Daping Hospital | Sputum | 0.6 | - |
| 29 | *Burkholderia cenocepacia* | Daping Hospital | Sputum | 0.8 | - |
| 30 | *Burkholderia cenocepacia* | Daping Hospital | Sputum | 1.6 | - |
| 31 | *Burkholderia cepacia* | Daping Hospital | Sputum | 1.6 | - |
| 32 | *Burkholderia cepacia* | Daping Hospital | BALF | 0.9 | - |
| 33 | *Burkholderia cepacia* | Daping Hospital | Sputum | 0.8 | - |
| 34 | *Burkholderia cepacia* | Daping Hospital | Sputum | 0.6 | - |
| 35 | *Pseudomonas aeruginosa* | Daping Hospital | Drainage | 0.6 | - |
| 36 | *Pseudomonas aeruginosa* | Daping Hospital | BALF | 1.6 | - |
| 37 | *Pseudomonas aeruginosa* | Daping Hospital | Sputum | 1.2 | - |
| 38 | *Pseudomonas aeruginosa* | Daping Hospital | Sputum | 0.8 | - |
| 39 | *Escherichia coli* | Daping Hospital | Secretion | 0.6 | - |
| 40 | *Escherichia coli* | Daping Hospital | Sputum | 0.8 | - |
| 41 | *Escherichia coli* | Daping Hospital | Sputum | 1.2 | - |
| 42 | *Escherichia coli* | Daping Hospital | Urine | 1.5 | - |
| 43 | *Klebsiella pneumoniae* | Daping Hospital | Urine | 0.6 | - |
| 44 | *Klebsiella pneumoniae* | Daping Hospital | BALF | 0.8 | - |
| 45 | *Klebsiella pneumoniae* | Daping Hospital | Urine | 0.6 | - |
| 46 | *Klebsiella pneumoniae* | Daping Hospital | Sputum | 0.9 | - |
| 47 | *Acinetobacter baumannii* | Daping Hospital | Sputum | 1.6 | - |
| 48 | *Acinetobacter baumannii* | Daping Hospital | BALF | 1.2 | - |
| 49 | *Acinetobacter baumannii* | Daping Hospital | Secretion | 1.3 | - |
| 50 | *Acinetobacter baumannii* | Daping Hospital | Secretion | 0.8 | - |

+: positive

-: negative


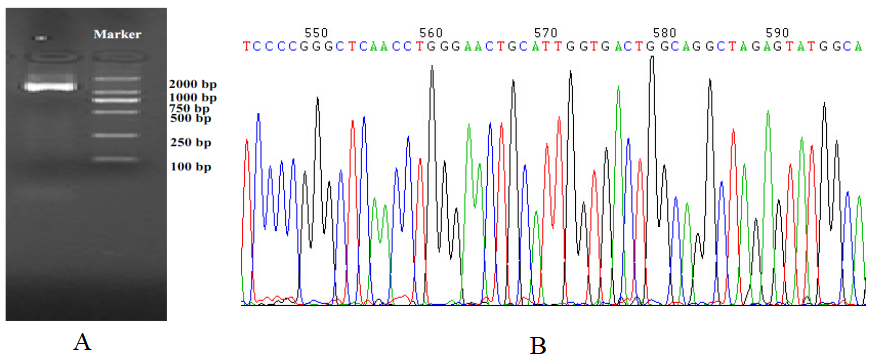


**Supplementary Figure 1. The *B. pseudomallei* isolate was retrospectively confirmed by a complete 16S rRNA gene sequencing analysis. A:** Electrophoretic map of PCR product of 16s rRNA gene**; B:** Sequencing of PCR product of 16s rRNA gene.

**
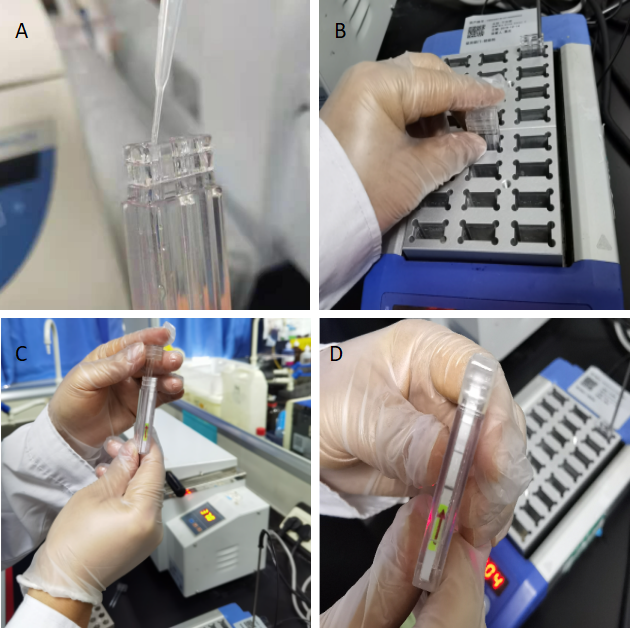
**

**Supplementary Figure 2.** **Scheme of the instant and visual methods for identifying *B. pseudomallei* based on recombinase polymerase amplification.** The operation steps were as follows: **A**, A 48 µL LF-RPA mix and 2 µL DNA sample were added in the reaction tube, and 190 µL buffer was added to another tube. **B**, The lid of the device was closed and the mixture was shaken for 5 seconds and incubate in a simple heating device at 40°C for 10 min. **C**, A probe was threaded through a small hole in the bottom of the tube and then the reaction and buffer were mixed thoroughly. **D**, Visual detection of the RPA amplicon with the help of the HybriDetect 1 lateral flow strip.
